# Prevalence of common mental disorders in mothers in the semiarid region of Alagoas and its relationship with nutritional status

**DOI:** 10.1590/S1516-31802012000200003

**Published:** 2012-04-03

**Authors:** Adriana Toledo de Paffer, Haroldo da Silva Ferreira, Cyro Rego Cabral, Claudio Torres de Miranda

**Affiliations:** I MSc. Nutritionist, Faculdade de Nutrição (Fanut), Universidade Federal de Alagoas (UFAL), Maceió, Alagoas, Brazil.; II PhD. Associate Professor, Faculdade de Nutrição (FANUT), Universidade Federal de Alagoas (UFAL), Maceió, Alagoas, Brazil.; III MD, PhD. Associate Professor, Faculdade de Medicina (FAMED), Universidade Federal de Alagoas (UFAL), Maceió, Alagoas, Brazil.

**Keywords:** Maternal welfare, Mental health, Urban population, Rural population, Nutritional status, Bem-estar materno, Saúde mental, População urbana, População rural, Estado nutricional

## Abstract

**CONTEXT AND OBJECTIVE:**

Compromised maternal mental health (MMH) is considered to be a risk factor for child malnutrition in low income areas. Psychosocial variables associated with MMH are potentially different between urban and rural environments. The aim here was to investigate whether associations existed between MMH and selected sociodemographic risk factors and whether specific to urban or rural settings.

**DESIGN AND SETTING:**

Cross-sectional study on a representative population sample of mothers from the semiarid region of Alagoas.

**METHODS:**

Multistage sampling was used. The subjects were mothers of children aged up to 60 months. MMH was evaluated through the Self-Reporting Questionnaire*-*20. Mothers’ nutritional status was assessed using the body mass index and waist circumference. Univariate analysis used odds ratios (OR) and chi-square. Logistic regression was performed separately for urban and rural subsamples using MMH as the dependent variable.

**RESULTS:**

The sample comprised 288 mothers. The prevalences of common mental disorders (CMD) in rural and urban areas were 56.2% and 43.8%, respectively (OR = 1.03; 95% CI: 0.64-1.63). In univariate analysis and logistic regression, the variable of education remained associated with MMH (OR = 2.2; 95% CI: 1.03-4.6) in urban areas. In rural areas, the variable of lack of partner remained associated (OR = 2.6; 95% CI: 1.01-6.7).

**CONCLUSIONS:**

The prevalence of CMD is high among mothers of children aged up to two years in the semiarid region of Alagoas. This seems to be associated with lower educational level in urban settings and lack of partner in rural settings.

## RESUMO

**CONTEXTO E OBJETIVO:**

Comprometimento da saúde mental materna (SMM) é considerado fator de risco para desnutrição infantil em áreas de baixa renda. Variáveis psicossociais associadas ao SMM são potencialmente diferentes em ambientes urbanos ou rurais. O objetivo foi investigar se existiria associação entre SMM e fatores de risco sócio-demográficos selecionados e se ela seria específica conforme o ambiente urbano/rural.

**TIPO DE ESTUDO E LOCAL:**

Estudo transversal, realizado com amostra representativa da população de mães do semi-árido alagoano.

**MÉTODOS:**

Amostragem em estágios múltiplos. Sujeitos foram mães de crianças até 60 meses de idade. SMM foi avaliada por meio do SRQ-20 (*Self-Reporting Questionnaire*). Estado nutricional materno avaliado por meio de índice de massa corporal e circunferência da cintura. Análise univariada utilizou OR (odds ratio) e qui quadrado. Regressão logística realizada separadamente com subamostras urbana e rural utilizando MMH como variável dependente.

**RESULTADOS:**

A amostra foi de 288 mães. Prevalência de transtornos mentais comuns foi 56,2% na zona rural e 43,8% na urbana (OR = 1,03; intervalo de confiança, IC 95%: 0,64-1,63). Tanto na análise univariada como na regressão logística, na área urbana, escolaridade foi a variável que permaneceu associada ao SMM (OR = 2,2; IC 95%: 1,03-4,6). Na zona rural a variável que manteve associação foi falta de parceiro (OR = 2,6; IC 95%: 1,01-6,7).

**CONCLUSÕES:**

A prevalência de transtornos mentais comuns é elevada entre as mães de crianças até dois anos de idade, na região semi-árida de Alagoas, o que parece estar associado com menor nível educacional no ambiente urbano e com a falta de um parceiro no ambiente rural.

## INTRODUCTION

Maternal mental health (MMH) is considered to be an important factor in the provision of adequate conditions for the growth and development of children, especially in low-income families.^1^ This is particularly relevant for mothers of children under two years of age, since the literature emphasizes the impact that maternal mental illness can have on children in this age group, with regard to both nutritional and developmental status.^2^^,^^3^^,^^4^^,^^5^^,^^6^^,^^7^^,^^8^^,^^9^^,^^10^^,^^11^ However, most of these studies have been conducted on urban populations.

Studies on low-income populations in developing countries have shown high prevalences of MMH problems.^12^^,^^13^^,^^14^ In a multicenter study conducted in four countries and involving mothers of children under two years of age, including both urban and rural areas, Harpham et al. found the following rates of MMH problems: 33.0% in Ethiopia, 30.0% in India, 30.0% in Peru and 21.0% in Vietnam. The instrument used by these authors to define outcomes of interest (common mental disorders, CMD) was the “Self-Reporting Questionnaire” (SRQ-20).^15^ In a study conducted on the outskirts of São Paulo, Brazil, de Miranda et al. reported that the frequency of CMD was 63% among mothers of malnourished children and 38% among mothers of normal children. These children were younger than two years of age.^5^ In an investigation carried out in Bahia, Brazil, among mothers of children under six years of age, Chagas found that the frequency of CMD was 35.8% among mothers of malnourished children and 20.1% among mothers of eutrophic children.^6^ In Rio de Janeiro, Brazil, Kac et al. found that the frequency of CMD was 54.2% among the mothers of children aged nine months of age.^16^

In a literature review on the characteristics of the distribution of mental disorders in the general population in both urban and rural environments, Judd et al. pointed out both the importance of and the difficulty in identifying the roles of urban and rural contexts as risk factors for mental disorders.^17^ In the opinion of these authors, the most important issue would be to identify which topics were specific or more important in the rural setting compared with the urban environment.

Few studies conducted in developing countries have examined the role of the difference between urban and rural settings, or stated how they could be characterized as risk factors associated with impaired mental health. Havenaar et al. compared the mental health of a rural population with an urban one in South Africa. They showed that in the urban context, being female and unemployed was associated with higher prevalence of CMD, while in the rural context, mental disorders were associated with lower educational status and belonging to a poorer family.^18^ Concerning Brazil, studies have only addressed either urban or rural environments in isolation. Among an urban female population in Bahia, the characteristics associated with CMD included being divorced/separated/widowed, having low educational status or income, having children, being the head of the family and not having time to devote to leisure activities.^19^

## OBJECTIVE

The aim of this study was to investigate whether there are any differences in selected sociodemographic risk factors between urban and rural environments, as well as to examine the association between maternal nutritional status and the presence of CMD, among mothers of children up to two years of age.

## METHODS

### Type of study

This was a cross-sectional population-based study that formed part of a larger project entitled “Nutrition and health of the mother/child population of the semiarid region of the Brazilian State of Alagoas”.

### Setting

This study was conducted in the urban and rural areas of the semiarid region of the State of Alagoas, Brazil. This region comprises 38 municipalities and has 884,668 inhabitants according to the census of 2000.^20^

### Sample

Sampling was carried out on the original study, in order to obtain a representative sample of children under five years of age living in the semiarid region of the state of Alagoas. A multistage sampling design was used, consisting of three stages: municipalities were randomly selected; sectors within each municipality were established; and one household was defined within each sector, from which consecutive households amounting to 944 mothers of children aged 0-5 years were selected. The selection of municipalities was done by systematic sampling with probability proportional to size. The subjects of this study comprised all the mothers in the sample of the larger study who had children up to two years of age, thus resulting in a sample size of 288 mothers.

### Procedures

The field team in charge of data collection consisted of two supervisors and ten interviewers, divided into two groups. All the information was gathered by trained interviewers between January and March 2007, and was obtained during visits to the selected households. The questionnaire used had been previously tested in a pilot study.

### Main measurements

### Anthropometric survey

The participants were weighed on a portable digital anthropometric scale, with a capacity of 150 kg and sensitivity of 100 g. Height was recorded in the standing position by using a stadiometer with accuracy of 1 mm. For data analysis, the criterion of body mass index (BMI) classification was used. BMI was calculated as the ratio between body mass (kg) and height (m) squared, following the World Health Organization (WHO) classification criteria of 1995:^21^ underweight (< 18.5 kg/m^2^), normal (between 18.5 and 24.9 kg/m^2^), overweight (between 25 and 29.9 kg/m^2^) and obese (³ 30 kg/m^2^). Waist circumference was measured with the aid of a tape measure with a precision of 1 mm. The cutoff point used was as recommended by WHO.^22^

### Maternal mental health (MMH)

MMH was evaluated through application of a validated screening questionnaire (Self-Reporting Questionnaire; SRQ-20)^15^ that was developed by WHO to be used as a screening tool for psychiatric disorders. It consists of 20 closed questions with two alternatives for the answers (yes/no). A cutoff ³ 8 positive responses was adopted to identify probable cases of CMD (sensitivity = 83% and specificity = 80%).^23^

### Sociodemographic variables

Selected variables were dichotomized for analysis purposes. Regarding employment status, mothers were categorized as homemakers or working mothers. The mothers’ schooling was categorized as insufficient (up to grade 4) or satisfactory (grade 5 or more). The mothers’ ages were categorized as up to 30 years or older than 30 years. 

Regarding socioeconomic status, the sample was divided into five classes,^24^ obtained through a score based on the head of household’s schooling level and ownership of certain household items.

### Statistical methods

For analysis purposes, the sample was divided in two subsamples according to residence status. This was defined according to location: urban, i.e. located in urban areas corresponding to cities, towns (district headquarters) or isolated urban areas; or rural, i.e. any designated area outside the urban area, including settlements, villages and clusters. These definitions of urban and rural areas followed the procedures used by the Brazilian Institute for Geography and Statistics (IBGE).^19^

In addition to MMH, 10 variables were dichotomized. (1) Mothers’ ages were divided into ? 30 years and > 30 years. This cutoff was chosen arbitrarily from the hypothesis that there is a change of social role for women in the social group from this age onwards. (2) Maternal educational level was defined as low when mothers had < 4 years of schooling and high when they had ³ 4 years of schooling. This cutoff was set because, in the past, four years was established by law as the duration of compulsory education, and many people were discontinuing school attendance at this point. Nowadays, the period considered fundamental by the Ministry of Education is a minimum of nine years. (3) The number of children per mother was divided into low, defined as up to three, and high, > 3. This cutoff was chosen because we believed that low-income families with > 3 children would have additional difficulties, such as more severe financial constraints and less time to spend with each child. (4) The parameter of government program refers to whether the individual was included or not in any government social programs. (5) BMI was stratified into two categories: eutrophic = BMI < 25 kg/m^2^ and overweight = BMI ³ 25 kg/m^2^, using the official cutoff point. (6) Marital status was described as with or without a partner (irrespective of legalized marriage). (7) The head of the family was defined as male or female. (8) Waist circumference of the mother was divided into < 80 cm and ³ 80 cm. (9) Work outside the home (paid work) by the woman was divided into yes and no. (10) Social class was defined according to the five classes proposed by the Brazilian Association of Polling Companies (Associação Brasileira de Empresas de Pesquisa; ABEP) and then dichotomized by bringing together classes A, B, and C as the higher classes and D and E as the lower classes. The low-income population studied consisted basically of classes C, D and E, and for this reason it was divided into C and D + E. The data were entered twice into the Epi Info spreadsheet version 3.3.2, and were analyzed with the aid of the Statistical Package for the Social Sciences (SPSS), version 14.0.

In the univariate analysis, the odds ratio (OR) with 95% confidence interval (CI) was used to determine the magnitude of the association. In each of the two multivariate logistic regressions (urban and rural), maternal CMD was used as the dependent variable, whereas the other variables were included as independent factors. All variables that were not significantly associated (P ≥ 0.1) were removed from the final models using the “backward” process. Correlations between variables were assessed using Pearson’s correlation coefficient (r ³ 0.70), in order to exclude autocorrelated variables from the analysis.

### Ethical issues

This research was approved by the Research Ethics Committee of Universidade Federal de Alagoas (UFAL) (Procedural No. 000465/2007 1996). Data were gathered only after the informed consent statement had been signed.

## RESULTS

Out of the sample of 288 women, 43.4% lived in urban areas and 56.6% lived in rural areas. Table 1 lists the socioeconomic, demographic and health characteristics of the study population.

The prevalences of CMD in the rural and urban area were 56.2% and 43.8%, respectively (OR = 1.03; 95% CI: 0.64 to 1.63).

In the rural area, there was a higher prevalence of mothers with less than four years of schooling (P = 0.001), a larger number of mothers from families receiving government financial support (P = 0.027) and a higher frequency of individuals belonging to lower social classes (P = 0.0001).

Table 2 depicts the association between the presence of CMD and the selected variables in the urban and rural environments. In the urban areas, there was significant association between CMD and maternal education (OR = 2.3; 95% CI: 1.08 to 4.8), thus showing that urban mothers with lower educational status were more likely to present CMD. No statistical difference was found between CMD and other variables. Among the rural women, the variable “without partner” was associated with a greater chance of having CMD (OR = 2.7; 95% CI: = 1.08 to 6.8). The association between presence of CMD and social class was not statistically significant (OR = 7.7; 95% CI: 0.96 to 6.3).

In both the rural and the urban logistic regression analysis, the following were considered to be independent variables: maternal age and schooling, working status, economic class, marital status, BMI, waist circumference and number of children. In the rural sample, the dependent variable (CMD) was associated with maternal schooling only (OR = 2.2; 95% CI: 1.3 to 4.6), whereas marital status was the only variable with statistical significance in the rural setting (OR = 2.6; 95% CI: 1.01 to 6.7). Therefore, the significant associations were the same as those found in the univariate analysis.

**Table 1.** Socioeconomic, demographic and maternal health characteristics in the semiarid region of Alagoas, 2007

Variables	Category	Urban (n = 125)	(%)	Rural (n = 163)	(%)	P
Common mental disorders	³ 8	56	43.8	72	56.2	0.91
	< 8	69	43.1	91	56.9	
Age (years)	15-25	67	45.6	80	54.4	0.61
	26-35	44	42.7	59	57.3	
	36-45	14	36.8	24	63.2	
Schooling (years)	< 4	45	32.8	92	67.2	0.001
	> 4	80	53.0	71	47.0	
Number of children	³ 3	39	36.4	68	63.6	0.067
	< 3	86	47.5	95	52.5	
Marital status (partner)	Yes	26	53.0	23	47.0	0.13
	No	99	41.4	140	58.6	
Gender of head of family	Male	101	42.8	135	57.2	0.65
	Female	24	46.2	28	53.8	
Government program	Yes	75	38.9	118	61.1	0.027
	No	50	52.6	45	47.4	
Work outside the home	No	54	45.7	64	54.3	0.5
	Yes	71	41.8	99	58.2	
Economic class (ABEP)	A	0		0		0.0001
	B	7	87.5	1	12.5	
	C	24	72.7	9	27.3	
	D	58	48.7	61	51.3	
	E	36	28.1	92	71.9	
Body mass index (kg/m2)	< 18.5	10	52.6	9	47.4	0.56
	18.5 - 24.99	64	40.0	96	60.0	
	25 - 29.99	34	47.9	37	52.1	
	³ 30	17	44.7	21	55.3	
Waist circumference (cm)	³ 80	50	46.7	57	53.3	0.38
	< 80	75	41.4	106	58.6	

ABEP = Associação Brasileira de Empresas de Pesquisa (Brazilian Association of Polling Companies).

**Table 2.** Association between maternal common mental disorders and selected variables in the urban and rural areas of the semiarid region of Alagoas, 2007

Variables	Category	Urban area (n = 125)						Rural area (n = 163)					
		SRQ						SRQ					
		Positive		Negative		OR	95% CI	Positive		Negative		OR	95% CI
		n	%	n	%			n	%	n	%		
Mother’s age (years)	³ 30	16	50.0	16	50.0	1.3	(0.6-2.9)	27	50.9	26	49.1	1.5	(0.7-2.9)
	< 30	40	43.0	53	57.0			45	40.9	65	50.1		
Maternal schooling level (years)	< 4	26	58.0	19	42.0	2.3	(1.08-4.8)	42	45.6	50	54.4	1.2	(0.6-2.1)
	> 4	30	37.0	50	63.0			30	42.3	41	57.8		
Number of children	³ 3	21	54.0	18	46.0	1.7	(0.8-3.6)	34	50.0	34	50.0	1.5	(0.8-2.8)
	< 3	35	41.0	51	59.0			38	40.0	57	60.0		
Marital status (partner)	Yes	12	46.0	14	54.0	1.1	(0.45-2.5)	15	65.2	08	34.8	2.7	(1.08-6.8)
	No	44	44.0	55	56.0			57	40.7	83	59.3		
Head of family	Male	43	42.6	58	57.4	0.6	(0.2-1.5)	61	45.2	74	54.8	1.3	(0.5-2.9)
	Female	13	54.0	11	46.0			11	39.3	17	60.7		
Government program	Yes	36	48.0	39	52.0	1.4	(0.7-2.8)	57	48.3	61	51.7	1.8	(0.9-3.8)
	No	20	40.0	30	60.0			15	33.3	30	66.7		
Work outside the home	No	21	39.0	33	61.0	0.6	(0.3-1.34)	23	35.9	41	64.1	0.6	(0.3-1.09)
	Yes	35	49.0	36	51.0			49	49.5	50	50.5		
Social class (ABEP)	D and E	44	47.0	50	53.0	1.4	(0.6-3.2)	71	46.4	82	53.6	7.7	(0.96-63)
	B and C	12	39.0	19	61.0			01	10.0	09	90.0		
Body mass index (kg/m2)	Overweight	26	51.0	25	49.0	1.5	(0.7-3.1)	26	45.6	31	54.4	1.1	(0.6-2.1)
	Eutrophic	30	40.0	44	60.0			46	43.4	60	56.6		
Waist circumference (cm)	³ 80	23	46.0	27	54.0	1.1	(0.53-2.2)	29	50.8	28	49.2	1.5	(0.8-2.9)
	< 80	33	44.0	42	56.0			43	40.6	63	59.4		

SRQ = Self Reporting Questionnaire; OR = odds ratio; CI = confidence interval; ABEP = Associação Brasileira de Empresas de Pesquisa (Brazilian Association of Polling Companies).

## DISCUSSION

The prevalences of CMD among mothers in the semiarid region of Alagoas were 43.8% and 56.3% in the urban and rural settings, respectively. In South Africa, Havenaar et al.^18^ found that the prevalences of CMD in men and women were 34.9% and 27.0% in urban and rural areas, respectively. By calculating the prevalence of CMD in the community only, without taking into account the subsamples treated in primary care and by traditional healers, these authors obtained prevalences of 43.3% and 41.9% among the female urban and rural populations, respectively. The sampling methodology used in the investigation by Havenaar et al.^18^ was quite different from that used in the present study. The data from their study have been presented here only to suggest that the prevalence of CMD among women in low-income populations in both urban and rural environments with other cultural contexts is also high.

Harpham et al. conducted a study in four countries, addressing the association between child malnutrition and the presence of maternal CMD. These authors used the variable urban/rural as only one of the intervening variables, and it did not present statistical significance.^2^ From the project website, it was possible to obtain the prevalences of CMD for both the urban and the rural environment subpopulations of each country surveyed. In Ethiopia, 33% of the mothers were positive according to the SRQ-20, both in urban and in rural settings;^25^ in India,^26^ Peru^27^ and Vietnam^28^ the prevalences were 32%, 32% and 39% in rural areas and 19%, 39% and 20% in urban areas, respectively.^25^ The prevalences encountered in the present study in both the urban and the rural areas were greater than those reported in the abovementioned countries. In comparisons between urban and rural environments, the high prevalence of cases of CMD among mothers in rural areas observed in our present investigation had only previously been observed in India. Among the possible reasons explaining the higher prevalence of maternal CMD in our study compared with the others, it is possible to envisage cultural specificities and occurrences of decreased social support in the region.

In Brazil, other cross-sectional studies on the prevalence of CMD have been performed in one environment only, i.e. either urban or rural. All the studies identified high prevalences of CMD. Araújo et al. found that the prevalence of mental disorders was 39.4% among women in an urban area of Feira de Santana, state of Bahia.^19^ Costa and Ludermir reported prevalences of 44.2% among women and 24.5% among men in a study conducted in rural areas of the state of Pernambuco.^29^ These results are consistent with those of the present study, thus indicating that the prevalence of CMD in women is very high both in urban and in rural environments.

According to Judd et al., urban-rural comparisons made between different studies do not take into account regional, cultural or methodological characteristics, thereby obscuring the existence of possible differences.^17^ Accordingly, comparisons between the data obtained in our study and data reported in the above authors’ review of the literature could be subject to this criticism.

In the studies included in the abovementioned review, which addressed the association of maternal CMD with sociodemographic risk factors and the nutritional status of mothers, the variable of urban/rural setting was included as just one of the risk factors investigated. Contrary to what is presented in our study, there was no mention of any study on the association between maternal CMD and other factors for the two contexts separately.

In the present study, low educational status was the only variable associated with the presence of maternal CMD in the urban area, whereas in rural areas, there was an association with lack of a partner. In a study on women in an urban area of the state of Pernambuco, Brazil, Ludermir and Melo Filho^30^ also found an association between CMD and poor education in a sample from a low-income population. In an investigation on women in urban areas in the state of Bahia, Brazil, Araújo et al. found an association between CMD and low educational level, namely no access to school (OR = 3.05; 95% CI: 1.9 to 5.02) or low schooling level (OR = 2.54; 95% CI: 1.57 to 4.10).^19^

In a study on a rural population conducted in the state of Pernambuco, the risk factors for CMD were female gender and, regardless of gender, separated or widowed status.^29^ In the study by Ludermir et al., also conducted in Pernambuco, an association between CMD and lack of a partner in the rural environment was also detected.^30^ In an urban study in Bahia, an association between CMD and poor education was also observed.^19^ The study in Pernambuco also reported that there was an association between mental disorders and low educational status in rural areas. This association was not observed in the present study.

Regarding maternal nutritional status in the present study, there was a high percentage of overweight and obesity, as well as of waist circumference larger than 80 cm, thus reflecting a high risk of metabolic complications associated with obesity. A similar result was also reported in the National Demographic and Health Survey,^31^ which investigated Brazilian women aged 15-49 years. In this survey, 42.8% and 44.7% of the women were overweight, and 16.1% and 15.8% were obese, in the urban and rural areas, respectively. Moreover, the prevalences of waist circumference larger than 80 cm were 52.2% and 52.8% in the urban and rural settings, respectively.^31^

Psychological problems such as anxiety and depression are also associated with weight gain, thereby influencing feeding behavior. However, in the present study, there was no association between CMD and waist circumference or BMI. In a survey of mothers of 9-month-old children, Kac et al. found a statistical association between CMD and body fat greater than or equal to 30% (OR = 1.66; 95% CI: 1.03 to 2.65). Nevertheless, there was no association between CMD and BMI, which is in agreement with the results from the present study. These authors stated that there was a paucity of studies correlating maternal nutritional status with CMD, and they suggested that further studies should be carried out.^16^

The association between MMH and their impact on the nutritional status of children has been identified by some authors in studies conducted in several countries. In the study by Harpham et al., conducted in four countries (Peru, Ethiopia, India and Vietnam), a significant relationship was found between MMH and nutritional status deficits in children in two countries (India and Vietnam).^2^ In a cohort study in Goa, India, Patel et al. showed that mothers with depression had a risk that was 2.3 times higher (95% CI: 1.1 to 4.7) of having children with malnutrition.^3^

In a case-control study conducted in Pakistan, Rahman et al. found that mothers of malnourished children had higher risk (OR = 3.9; 95% CI: 1.95 to 7.86) of presenting mental disorders than did mothers of normal children.^4^ In Brazil, a case-control study conducted in São Paulo by de Miranda et al. found that mothers of malnourished children had a higher risk (OR = 2.8; 95% CI: 1.2 to 6.9) of presenting emotional problems than did mothers of normal children.^5^

In a prospective study conducted by the same group in Pakistan, Rahman et al. estimated that reducing the prevalence of maternal depression could reduce the growth retardation in children by up to 30%.^32^ Therefore, intervention programs to improve maternal mental health should be considered as a strategy for combating child malnutrition.^12^ Nevertheless, the mother’s mental health is neglected by most child health programs in developing countries.^2^

One major limitation of our study was that its cross-sectional design did not allow any hypothesis regarding the direction of causality between the associations that were found, namely between mental disturbances and schooling in urban areas, and between CMD and lack of a partner in rural contexts. Another limitation was that no structured diagnostic instrument allowing psychiatric diagnoses was used. A further limitation was the small number of malnourished children identified, which led to reduction of the power of the study.

Regarding implications for future research, there is a need for prospective studies in order to provide more credibility to these hypotheses. It would be also useful to conduct trials in both urban and rural areas to test whether schooling would have a different impact in each of them. Qualitative studies could provide a better understanding of the possible associations evaluated. Another implication of this study is that it raises maternal mental health as an important public health topic to be addressed in this region. Regarding further research, the present study points out the importance of MMH as an important topic for studies addressing reduction of disease burden. Since the findings from this study are in accordance with the results from studies conducted in other regions of Brazil, as well as in other countries around the world, it can be said that the conclusions can be generalized.

## CONCLUSION

In accordance with other studies on low-income populations, the prevalence of CMD is high among the mothers of children up to two years of age in the semiarid region of Alagoas. This seems to be associated with lower educational status in the urban environment and with the lack of a partner in the rural areas.

Besides giving evidence of maternal nutritional problems, the results from this study suggest that there is a need to providing resources to cope with the high prevalence of CMD among mothers of young children, in order to reduce the damage to both the mother and the child under her responsibility. Furthermore, the present investigation suggests that there is a need for prospective studies that could prove the specificity of the associations between the identified risk factors and maternal mental health in the urban and rural environments.
